# Profil épidémiologique de la dermatomyosite et de la polymyosite: expérience du service de rhumatologie de Marrakech

**DOI:** 10.11604/pamj.2021.38.101.25406

**Published:** 2021-01-29

**Authors:** Souhil Errafia, Ahmed Mougui, Imane El Bouchti

**Affiliations:** 1 Service de Rhumatologie, Centre Hospitalier Universitaire Mohammed VI, BP 2360 principal, Avenue Ibn Sina, Marrakech, Maroc

**Keywords:** Polymyosite, dermatomyosite, épidémiologie, pneumopathie interstitielle, cancer, Polymyositis, dermatomyositis, epidemiology, interstitial lung disease, cancer

## Abstract

La dermatomyosite (DM) et la polymyosite (PM) sont des affections rares mais graves. Le but de ce travail est d'étudier, à travers la revue d'une série hospitalière, leur profil épidémiologique, clinique et évolutif. Il s´agit d´une étude rétrospective, conduite sur une période de 15 ans, entre janvier 2004 et décembre 2019. Ont été retenus les cas où le diagnostic était certain ou probable selon les critères de Bohan et Peter. Un total de 14 patients ont été inclus (8 DM et 6 PM), l´âge moyen était de de 48,7 ans. La sex-ratio était de 13F/1H. Les signes généraux existaient dans 71%. Le déficit moteur touchait les muscles des ceintures dans 71% des cas; 85,7% avaient des arthralgies et 14% des arthrites. L´érythème et l´œdème périorbitaire étaient les signes cutanés prédominants. Au bilan, la vitesse de sédimentation était accélérée chez tous les malades et les enzymes musculaires augmentées dans 80%. Les anticorps anti-nucléaires étaient positifs dans 63%. La biopsie musculaire a retrouvé une myosite inflammatoire dans 75%. L'atteinte cardiaque existait dans 14% des cas et pulmonaire dans 21%. Un cancer était associé dans 21,4% des cas. Tous les patients ont reçu une corticothérapie. L´évolution s´est faite vers l´amélioration dans 88%, avec une rechute chez 4 patientes. Dans notre contexte, la DM est plus fréquente que la PM, avec nette prédominance féminine. L'atteinte pulmonaire reste une complication lourde. L´association aux cancers semble fréquente d´où la nécessité d´un bilan systématique de néoplasie au moment du diagnostic et lors du suivi.

## Abstract (en)

Dermatomyositis (DM) and polymyositis (PM) are rare but serious conditions. The purpose of this study was to investigate, by a review of hospital cases, their epidemiological, clinical and evolutionary profile. We conducted a retrospective study over a 15-year period, between January 2004 and December 2019. All cases with possible or definite diagnosis according to Bohan and Peter’s criteria were retained. A total of 14 patients were enrolled (8 DM and 6 PM), with an average age of 48.7 years. Sex ratio was 13F/ 1H. General signs were reported in 71% of cases. Motor deficit affected the girdle muscles in 71% of cases; 85.7% of patients had arthralgia and 14% arthritis. Erythema and periorbital edema were the predominant skin signs. Patients’ assessment showed increased sedimentation rate in all cases and increased muscle enzymes in 80% of cases. Antinuclear antibodies were positive in 63% of cases. Muscle biopsy objectified inflammatory myositis in 75% of cases. Heart disease was reported in 14% of cases and lung disease in 21%. Cancer was found in 21.4% of cases. All patients received corticosteroid therapy. Improvement was reported in 88% of patients, with a relapse in 4 patients. In our context, DM is more frequent than PM, with a clear female predominance. Pulmonary disease is a heavy complication. Its association with cancers occurs commonly, hence the need for a systematic cancer screening at diagnosis and follow-up.

## Introduction

Les polymyosites (PM) et dermatomyosites (DM) sont des connectivites rares, d´étiologie mal connue, dotées d´un grand polymorphisme clinique et évolutif. Elles appartiennent au groupe des myopathies inflammatoires idiopathiques caractérisées par une inflammation chronique des muscles striés [1]. Les PM sont caractérisées par une atteinte primitive endomysiale de mécanisme cellulaire cytotoxique (lymphocytes T-CD8), alors que les DM sont caractérisées par une atteinte primitive périvasculaire de mécanisme humoral (lymphocytes B) [2]. Sur le plan clinique, La faiblesse musculaire est le signe d'appel habituel et les lésions cutanées sont souvent typiques dans la DM. Pratiquement, le diagnostic se base sur un ensemble d´arguments cliniques, biologiques, immunologiques, électriques et histologiques. Le traitement fait appel classiquement aux corticostéroïdes. Cependant, l´adjonction d´un immunosuppresseur adjuvant est fréquente [1,2]. Les données concernant ces affections sont encore rares. Le but de notre travail est d´étudier les caractéristiques épidémiologiques, clinico-paracliniques et évolutives des PM et DM au service de rhumatologie et de les comparer aux données de la littérature.

## Méthodes

Il s´agit d´une étude rétrospective descriptive, analysant les dossiers des malades hospitalisés au service de rhumatologie sur une période de 15 ans, de janvier 2004 à décembre 2019. Ont été inclus les patients présentant une PM (certaine ou probable) ou une DM (certaine ou probable) selon les critères de Bohan et Peter 1975 (Tableau 1).

**Tableau 1:** critères diagnostiques de Bohan et Peter

A. Déficit musculaire bilatéral et symétrique des ceintures scapulaire et pelvienne
**B**. Elévation du taux sérique des enzymes musculaires (créatinine-phosphokinase)
**C**. Triade caractéristique à l´électromyogramme: potentiel d´unité motrice court et polyphasique, fibrillation et décharge répétée à haute fréquence.
**D**. Biopsie musculaire d´un muscle proximal: aspects caractéristiques; nécrose des fibres musculaires, foyers de régénération et infiltrats inflammatoires mononuclées, atrophie....
Manifestations cutanées caractéristiques:
**a)** Erythème périorbitaire en lunette prédominant sur les paupières supérieures
**b)** Et/ou un érythème douloureux, squameux de la sertissure des ongles ou de la face d´extension des articulations
Le diagnostic de polymyosite est affirmé avec certitude par la présence de 3 des 4 premiers critères, le diagnostic de dermatomyosite nécessite en plus la présence de signes cutanés.

Une PM certaine a été définie par la présence de 4 critères (ABCD), une PM probable a été définie par l´existence de 3 critères sur 4 (ABCD). Une DM certaine a été définie par la présence de 5 critères (ABCDE), une DM probable a été définie par l´existence de 3 critères sur 4 (ABCD) en plus des signes cutanés (E) [3]. L´atteinte pharyngée a été retenue devant l´existence de fausses routes ou de dysphagie avec une endoscopie œsophagienne normale ou montrant un spasme sphinctérien (en absence d´une pathologie tumorale ou d´autres diagnostics différentiels). L´atteinte laryngée a été recherchée à l´interrogatoire par l´existence d´une dysphonie. Le dosage de la créatine phosphokinase (CPK) a été considéré élevé quand il était supérieur à 1,5 fois la normale du laboratoire.

La norme du lactate déshydrogénase (LDH) était < 40 U/L et des Aldolases comprise entre 22-59 mU/L [4]. L´atteinte pulmonaire a été recherchée systématiquement par une radiographie thoracique, complétée en cas de signes cliniques ou radiologiques par une exploration fonctionnelle respiratoire (EFR) et/ou une tomodensitométrie. L´atteinte cardiaque a été recherchée en effectuant un électrocardiogramme (ECG), complété éventuellement par une échographie transthoracique (ETT). La recherche d´un cancer associé comprenait l´examen clinique, la radiographie thoracique, l´échographie abdominopelvienne, thyroïdienne et mammaire. Le traitement et l´analyse des données ont été réalisés avec toutes les garanties de confidentialité.

## Résultats

Au total, 14 malades ont été inclus. L´âge moyen était de 48,71 ans [17-74]: 41,6 ans [17-62] pour le groupe DM et 58,2 ans [28-74] pour le groupe PM. Le sexe-ratio était de 13/1 (7/1 pour la DM et 6/0 pour la PM). La répartition des cas selon des tranches d´âges de dix ans, montrait un pic de fréquence des DM entre 31 et 40 ans et 2 pics de fréquence des PM entre 51 et 70 ans.

Le tableau clinique était caractérisé par une symptomatologie générale, musculaire, articulaire et cutanée riche et variée (Tableau 2).

**Tableau 2:** répartition selon les signes cliniques

Signes cliniques		DM (n=8) n (%)	PM (n=6) n (%)	Total (n=14) n (%)
**Signes généraux**	Asthénie	2 (25)	1 (17)	3 (21)
	Fièvre	4 (50)	4 (67)	8 (57)
	Amaigrissement	2 (25)	4 (67)	6 (43)
**Signes musculaires**	Myalgies	8 (100)	6 (100)	14 (100)
	Déficit musculaire proximal	5 (62,5)	5 (83)	10 (71)
	Atteinte pharyngée	1 (12,5)	1 (17)	2 (14)
	Atteinte laryngée	0	0	0
**Signes articulaires**	Arthralgies inflammatoires	7 (87,5)	5 (83)	12 (86)
	Polyarthrite chronique non déformante	1 (12,5)	1 (17)	2 (14,2)
**Signes cutanés**	Œdème du visage	4 (50)	-	-
	Erythème (périorbitaire ou péri-unguéal)	8 (100)	-	-
	Papule de Gottron	2 (25)	-	-
	Télangiectasies	1 (13)	-	-
	Syndrome de Raynaud	0	-	-
	Calcification sous cutanées	2 (25)	-	-

**Signes généraux:** dix malades (71%) ont présenté des signes généraux, à type d´asthénie, de fièvre ou d´amaigrissement.

**Signes musculaires:** les myalgies ont été rapportées chez tous les patients, prédominantes aux muscles proximaux. Le déficit musculaire, concernait 10 malades (71%), intéressant les ceintures pelviennes et scapulaires. Deux patientes avaient une atteinte pharyngée comprenant une dysphagie et des fausses routes alimentaires. Quant à l´atteinte laryngée, aucun malade n´a rapporté une dysphonie.

**Signes articulaires:** des arthralgies de type inflammatoire étaient rapportées chez 12 malades (85,7%), touchant essentiellement les grosses et les moyennes articulations périphériques. Des arthrites non déformantes étaient observées chez 2 patients (14,2%).

**Signes cutanés:** les signes qui ont été identifiés chez les patients DM: érythème périorbitaire, péri-unguéal ou aux faces d´extension articulaires (100%), œdème du visage (50%), papules de Gottron (25%) et calcifications sous cutanées (25%).

Les résultats des examens complémentaires réalisés sont contenus dans le Tableau 3.

**Tableau 3:** répartition en fonction des résultats des paramètres paracliniques

Signes paracliniques		DM (n=8) n (%)	PM (n=6) n (%)	Total (n=14) n (%)
**Marqueurs inflammatoires**	Vitesse de sédimentation accélérée	8/8 (100)	6/6 (100)	14/14 (100)
**Enzymes musculaires**	CPK augmentées	6/8 (75)	5/6 (83,3)	11/14 (78,5)
	LDH augmentés	7/8 (87,5)	3/4 (75)	10/12 (83,3)
	Aldolases augmentées	0	2/3 (66,6)	2/3 (66,6)
**Bilan immunologique**	Ac-Antinucléaires positifs	4/7 (57,1)	3/4 (75)	7/11 (63,6)
	Ac Anti-Jo1 positifs	1/7 (14,2)	3/4 (75)	4/11 (36,3)
	Ac Anti-SSA positifs	2/7 (28,5)	1/4 (25)	3/11 (27,2)
**Electromyogramme**	Tracé myogène	1/3 (33,3)	4/4 (100)	5/7 (71,4)
	Tracé neurogène	1/3 (33,3)	0/4 (0)	1/7 (14,2)
	Tracé normal	1/3 (33,3)	0/4 (0)	1/7 (14,2)
**Biopsie musculaire**	Myosite interstitielle avec des foyers de nécrose, atrophie et régénération	3/5 (60)	3/3 (100)	6/8 (75)
	Myosite non spécifique	2/5 (40)	0/3 (0)	2/8 (25)
**Signes cardio-pulmonaires**	Bloc de branche droit	2/8 (25)	0/6 (0)	2/14 (14,2)
	Pneumonie interstitielle diffuse	3/8 (37,5)	0/6 (0)	3/14 (21,4)

**Marqueurs inflammatoires:** tous les patients avaient une vitesse de sédimentation (VS) accélérée, avec une moyenne estimée à 55 mm/heure.

**Enzymes musculaires:** la créatine phosphokinase (CPK) était augmentée de plus de 1,5 fois la normale dans 78,6% des cas avec une moyenne de 530 UI. Les Lactates déshydrogénases ont été mesurés chez 12 patients, augmentés de plus de 1,5 fois la normale dans 83% des cas avec une moyenne de 350 UI. Le dosage des Aldolases n´a été effectué que chez 3 patients. Ils étaient élevés dans 2 cas.

**Bilan immunologique:** le dosage des anticorps antinucléaires (AAN) était réalisé chez 11 patients: positifs chez 7 patients, de type moucheté avec des anti-Jo1 positifs chez 4 patients et Anti SSA positifs chez trois; négatifs chez 4 patients.

**Electromyogramme (EMG):** il a été réalisé chez 7 patients. Il montrait un tracé de type myogène chez 5 cas (71%), neurogène dans un cas (14%) et jugé normal dans un cas (14%).

**Biopsie musculaire:** réalisée chez 8 malades, elle était en faveur d´une myosite inflammatoire (muscle siège d´un infiltrat inflammatoire interstitiel avec présence de zones de nécrose, d´atrophie et de foyers de régénération) dans 6 cas (75%) et non concluante (muscle siège de réaction inflammatoire non spécifique) chez les deux restants (25%).

**Bilan de retentissement cardio-pulmonaire:** il a été effectué chez tous les patients objectivant que deux cas de DM (14%) avaient un bloc de branche droit (BBD); trois cas de DM (21%) avaient une pneumopathie interstitielle diffuse (PID) radiographique dont une a été confirmée par un scanner.

Les cancers et les maladies de systèmes étaient les principales pathologies associées à la myosite inflammatoire (Tableau 4).

**Tableau 4:** répartition en fonction des comorbidités

Comorbidités		DM (n=8) n (%)	PM (n=6) n (%)	Total (n=14) n (%)
**Cancers**	Cancer du sein	2 (25)	0	3 (21,4)
	Cancer thyroïdien	0	1 (17)	
**Maladies de système**	Lupus	1 (12,5)	0	3 (21,4)
	Polyarthrite rhumatoïde	0	1 (17)	
	Sclérodermie	1 (12,5)	0	

La corticothérapie était indiquée en première intention chez tous les patients et administrée à forte dose (0,5 à 1 mg/kg/j de prednisone). Elle a été initiée par des bolus de méthylprednisolone à raison de 0,25 à 1g/j trois jours de suite chez 10 patients. Le méthotrexate à la dose de 15 mg/semaine a été indiqué chez deux patientes: une PM associée à une polyarthrite rhumatoïde et une DM corticodépendante.

La durée moyenne du suivi était de 4 ans (6-72 mois). L´évolution s´est fait vers la rémission des symptômes et la normalisation des CPK dans 86% des cas après 6 mois de traitement. Une rechute a été signalée chez 4 patientes DM, une corticodépendance chez un cas de DM, et 6 patients ont été perdus de vue après 1 année de suivi. Aucune néoplasie incidente n´a été notée durant le suivi et aucun décès n´a été déploré.

## Discussion

Une prépondérance des cas de DM est notée dans cette série (57% DM vs 43% PM). Cette prépondérance est également observée dans la série espagnole de Selva-O´Callaghan *et al*. (70% DM et 30% PM) et la série tunisienne de S. Toumi *et al*. (71% DM et 29% PM) [5,6]. L´exposition aux rayons ultraviolets (UV) pourrait jouer un rôle dans le déclenchement de la DM comme cela a été suggéré par l´étude d´Okada *et al*. où la proportion des cas de DM dans un groupe de myosites inflammatoires était fortement corrélée à l´intensité de l´irradiation par les UV par surface, exprimée en joules par mètre carré [7]. Ainsi, dans la région de Mexico, 79% des myosites étaient des DM, alors que dans la région de Glasgow, seulement 27% des myosites étaient des DM [6]. La prédominance féminine constatée est retrouvée dans toutes les séries de la littérature, le sex-ratio femme/homme varie entre 1,5 et 4 [5,8,9]. Le rôle des hormones sexuelles dans l´exacerbation de la maladie a été discuté, tenant compte que cette prédominance affecte plutôt les femmes en âge de procréer, l´aggravation fréquente de la maladie lors de la grossesse, aussi qu´un plus grand risque de complications materno-fœtales par rapport à la population générale [10]. Concernant les manifestations musculaires, les résultats sont concordants avec ceux de la littérature: l´atteinte des ceintures, varie de 70 à 100% dans la littérature, l´atteinte de la musculature pharyngée de 10 à 45% et l´atteinte laryngée reste plus rare (0-5%) [6,8,9,11].

L´érythroedème des paupières est le signe cutané de DM le plus fréquent. Il s´agit d´un érythème lilacé accompagné d´œdème touchant les paupières et pouvant s´étendre aux joues, sans atteindre la racine du nez contrairement à l´érythème du lupus. L´érythème peut toucher également la région péri-unguéale donnant ainsi le signe de la manucure évocateur de la maladie; Il s´agit d´un érythème congestif, rouge vif et douloureux à la pression. L´érythème peut s´étendre également au décolleté, face d´extension des genoux, coudes et mains [6,8,9,12,13]. Nous avons constaté une différence entre les deux groupes pour la survenue de rhabdomyolyse (élévation des CPK, LDH, Aldolases, Transaminases): celle-ci est plus fréquente en cas de PM, témoignant probablement d´une atteinte musculaire constante. S. Toumi *et al*. [6] aussi que Scola *et al*. [8] ont d´ailleurs trouvé un taux de CPK significativement plus élevé au cours des PM, témoignant d´une rhabdomyolyse plus intense.

La fréquence de l´atteinte cardiaque est diversement appréciée dans la littérature et varie entre 6 et 75% des cas selon les moyens d´investigation utilisés pour sa recherche [14]. Les troubles électriques sont les plus fréquents: arythmies, blocs de branche droit (BBD), blocs auriculoventriculaires et anomalies du segment ST [8,11,15]. L´atteinte myocardique a été également rapportée sous forme de dysfonction diastolique du ventricule gauche, d´hyperkinésie ventriculaire ou de défaillance cardiaque [16,17]. Dans notre série 14% avaient un BBD. Il ne semble pas y avoir de facteurs cliniques ou biologiques prédictifs de l´atteinte cardiaque d´après l´étude prospective de Taylor *et al*. [16]. Parmi les atteintes viscérales spécifiques, la pneumopathie interstitielle diffuse (PID) est l´atteinte la plus fréquente, présente chez 21,4% des cas de notre série. Sa fréquence est variable dans les études, elle est évaluée à 39% à l´étude de Selva-O´Callaghan *et al*. qui n´a pas constaté de particularités cliniques ou épidémiologiques dans le groupe ayant une PID par rapport à celui qui en est exempt [5]. La présence d´anticorps anti-synthétases est considérée, par ailleurs, comme un facteur prédictif de survenue de PID. Cette dernière, peut survenir à n´importe quel moment de l´évolution de la myosite [5,18,19]. Elle peut se manifester par une dyspnée à l´effort, accompagnée ou non de toux sèche, d´installation progressive ou aiguë. Elle peut aussi rester asymptomatique d´où la nécessité de la rechercher systématiquement par la pratique d´un scanner thoracique à haute résolution et des explorations fonctionnelles respiratoires [20,21]. Elle est considérée comme un facteur de mauvais pronostic car souvent responsable d´une lourde mortalité par insuffisance respiratoire et justifie un traitement immunosuppresseur [22,23].

L´association de la PM ou DM à un cancer varie entre 6 et 40% des cas selon les études [9,11,12,15,22-29]. L´existence d´un lien non fortuit entre néoplasie et myosite est désormais bien établie, le risque de survenue de cancer en cas de DM étant plus élevé qu´en cas de PM [22-25]. Le diagnostic de néoplasie est dans la majorité des cas concomitant à celui de la myosite ou fait quelques mois plus tard. Cependant, une persistance du risque jusqu´à deux ans après le diagnostic de DM a été signalée [30]. Certains facteurs sont considérés comme prédictifs de survenue de cancers tels qu´un âge de début tardif supérieur à 50 ans, des signes cutanés et musculaires très marqués, la présence de nécrose cutanée, la présence de signes généraux ou un taux de CPK très élevé [28-31]. La présence d´une PID est un facteur plutôt protecteur [27,28]. Les néoplasies les plus fréquemment associées aux PM et DM seraient en fait celles les plus endémiques dans la population concernée. Dans notre série, nous avons une prédominance du cancer du sein et de la thyroïde; La même chose était objectivée par S. Toumi *et al*. qui a trouvé dans sa série la prédominance du cancer du sein chez les femmes DM et le cancer du cavum chez les hommes DM [6].

Pour rechercher une néoplasie chez un patient présentant une PM ou une DM, la majorité des auteurs préconisent de se limiter à un examen clinique complet avec touchers pelviens. Une tomodensitométrie thoraco-abdominale et/ou une colonoscopie sont à pratiquer chez les patients présentant des facteurs prédictifs de néoplasie [29,32]. On peut proposer en plus de ces recommandations la pratique régulière d´un examen ORL chez un patient provenant d´un pays endémique pour les néoplasies du nasopharynx (Maghreb et Asie), une échographie cervicale, mammographie et une échographie abdomino-pelvienne.

Dans notre série, tous les patients ont reçu comme traitement de première ligne une corticothérapie de forte dose avec une amélioration satisfaisante. Le recours à un immunosuppresseur (Méthotrexate) dans notre cas a été instauré devant la corticodépendance dans un cas et devant l´association à une polyarthrite rhumatoïde dans l´autre. Cela concorde avec la prise en charge proposée dans la littérature. En effet, on recommande d´adjoindre à la prednisone (0,5 à 1 mg/kg/jour) au long cours un traitement d´entretien à la base de méthotrexate (7,5 à 20 mg/semaine), d'azathioprine (2 à 3 mg/kg/j) ou de ciclosporine A (2 à 3 mg/kg/j) en cas d´une forme réfractaire à la corticothérapie, dans les formes viscérales graves ou parfois pour l´épargne cortisonique. La cyclophosphamide est réservée aux atteintes pulmonaires interstitielles aigues [33].

Le pronostic des PM et DM est globalement péjoratif en raison d´une morbidité élevée et d´une mortalité importante. En effet, la guérison complète ne peut être obtenue que dans peu de cas et la stabilisation s´accompagne souvent de déficit moteur persistant, responsable d´handicap: 50% seulement des patients en rémission retrouvent leur niveau d´activité physique antérieure d´après l´étude de Marie *et al*. [34]. Le décès, reste estimé entre 11 à 33% des cas dans la littérature, est dû essentiellement à l´atteinte respiratoire (insuffisance respiratoire aiguë et pneumopathie de déglutition) et à l´atteinte cardiaque (insuffisance cardiaque et troubles du rythme) [6]. Les différents facteurs prédictifs de mortalité retrouvés par certaines études sont l´atteinte pulmonaire (surtout la PID au cours de la DM), l´atteinte cardiaque, l´atteinte œsophagienne, l´âge avancé et la présence de néoplasie [5, 34-38].

## Conclusion

Les myopathies inflammatoires de type PM et DM sont des maladies de système qui restent rares dans notre contexte. Malgré le caractère rétrospectif et la faible taille d´échantillon, cette série nous a permis de mieux connaître les caractéristiques de ces connectivites. Nos résultats sont comparables à ceux de la littérature en ce qui concerne l´âge, la prédominance féminine, la fréquence des atteintes pulmonaires et cardiaques, ainsi que l´association possible à des cancers qui doivent être recherchés systématiquement après le diagnostic. Certaines particularités doivent être soulignées, notamment la prépondérance des DM par rapport aux PM qui semble être commune aux pays ensoleillés, un âge de début relativement jeune, et une incidence élevée des cancers du sein et de la thyroïde probablement due à son caractère endémique au Maroc. En général, ces maladies sont responsables d´une importante morbidité et mortalité malgré le traitement intensif par corticoïdes et immunosuppresseurs dont les effets indésirables alourdissent le pronostic de la maladie.

### Etat des connaissances sur le sujet

- Les dermatopolymyosites sont des connectivites rares;
- Les dermatopolymyosites sont dotées d´un polymorphisme clinique et évolutif;
- Les dermatopolymyosites sont fréquemment associées aux cancers.

### Contribution de notre étude à la connaissance

- Les dermatopolymyosites doivent chercher toujours un cancer associé, notamment du sein ou de la thyroïde;
- La corticothérapie demeure un traitement de première ligne efficace pour les dermatopolymyosites.
